# Growth mindset and socioeconomic inequality in academic achievement across seventy-three PISA countries

**DOI:** 10.1038/s41539-025-00365-8

**Published:** 2025-11-20

**Authors:** Joan E. Madia, Rob J. Gruijters, Isabel J. Raabe, Nicolas Hübner

**Affiliations:** 1 University of Oxford, Oxford, UK; 2 University of Bristol, Bristol, UK; 3 University of Zurich, Zurich, Switzerland; 4 University of Bonn, Bonn, Germany

**Keywords:** Social sciences, Education

## Abstract

Growth mindset is widely perceived to be a powerful lever for reducing inequalities in learning outcomes. This study investigates whether a growth mindset moderates or mediates the effect of socio-economic status (SES) on academic achievement across 73 countries, using data from the 2022 Programme for International Student Assessment (PISA). To do this, we employ a four-way decomposition approach to separate the total effect of SES on standardized test scores in math, reading, and science into direct, indirect, and interaction effects. The results show that growth mindset mediates only a small portion of the effect of SES on student achievements, accounting for no more than 2.9% to 3.2% of the total effect, depending on the subject. These findings challenge the influential idea that growth mindset can ‘temper’ the effect of poverty on academic achievement.

## Introduction

In recent years, there has been growing interest in understanding the role of growth mindset in shaping educational outcomes, particularly in the context of socioeconomic inequality. Growth mindset, the belief that intelligence is malleable rather than fixed, has emerged as a prominent concept in educational psychology^1^. Empirical evidence suggests that students who hold a growth mindset tend to achieve higher levels of educational attainment compared to those with a fixed mindset (e.g^2^.) and experimental studies show that even brief growth mindset intervention can lead to lasting improvements in academic performance^3^.

Moreover, several studies suggest that growth mindset is particularly beneficial for low-achieving and/or socioeconomically disadvantaged populations, and can therefore serve to reduce educational inequalities (see^4^). One particularly influential study, cited >1400 times to date and downloaded almost 200,000 times, claimed that having a growth mindset “tempers the effects of poverty on achievement” in Chile^5^. These findings have informed the widespread adoption of growth mindset interventions and curricula in schools in the US and elsewhere, often with the explicit aim of reducing educational inequality. This study, however, does not quantify the extent to which growth mindset mediates or moderates the effect of growth mindset on student achievement.

In addition, while cross-country studies have provided valuable insights into the role of growth mindset in diverse contexts, they have often focused on different aspects of this relationship. For instance, Bernardo et al.^6^ examined how societal beliefs influence the general association between growth mindset and achievement across cultures. Kaya et al.^7^ explored the relationship between growth mindset and achievement among immigrant students in specific countries. These studies offer important perspectives on cross-cultural variations and specific population effects. However, they do not directly investigate the extent to which growth mindset mediates the effect of socioeconomic status on academic achievement across a broad range of countries. Our study addresses these gaps by employing a four-way decomposition approach to quantify the mediating role of growth mindset in the SES-achievement link using data from 73 countries, providing a more comprehensive understanding of this relationship at a global scale.

More precisely, in this study, we seek to assess the extent to which growth mindset ‘tempers’ or ‘buffers’ the effect of socioeconomic origins on academic achievement, as claimed by Claro et al.^5^ and others. To do so, we first define the relevant theoretical and empirical estimands^8^. We then proceed to test the hypothesized relationships between family socioeconomic status (SES), growth mindset, and academic achievement in a counterfactual decomposition framework^9–11^. Our framework distinguishes between three types of effects: direct (capturing the effect of SES net of growth mindset), indirect (capturing the effect of SES that operates through growth mindset), and interactive (capturing how the SES effect varies by growth mindset)^8,10,11^.

In contrast to previous studies, which often rely on data from a single country, we use newly available data from the 2022 Programme for International Student Assessment (PISA) dataset covering 73 countries^12^. By examining the nuanced relationships between SES, growth mindset, and academic achievement across several countries, our study contributes to a deeper understanding of the mechanisms underlying socioeconomic inequality in educational outcomes.

The number of meta-analyses on the effects of growth mindset interventions on students’ academic achievement is on the rise. Two recent issues of the leading journal *Psychological Bulletin* were devoted entirely to the effects of growth mindset interventions on student outcomes (e.g., academic achievement), including two meta-analyses, three commentaries, and one reply on this topic. The findings from the two meta-analyses by Burnette et al.^4^ and Macnamara and Burgoyne^13^ show that the current state of research on the effects of growth mindset interventions on student achievement is mixed.

Burnette et al.^4^ examined 53 distinct samples and interventions. Based on this data, the authors found growth mindset interventions to positively affect academic achievement (*d* = 0.14, Cohen 1988). Notably, the authors also reported that future interventions would likely vary greatly in their effectiveness, as 95% prediction intervals ranged from *d* = -0.08 to *d* = 0.35. They also found that the intervention effects turned out to be larger for students in specific focal groups (e.g., at-risk students). In contrast to these findings, Macnamara and Burgoyne^13^ reported results based on three meta-analyses, considering 79 distinct samples. When selecting studies, the authors particularly focused on those that considered best practices for drawing causal conclusions. Overall, Macnamara and Burgoyne^13^ found a small average effect of growth mindset interventions on student achievement of *d* = 0.05 that did not reach statistical significance after correcting for publication bias. Interestingly, the authors did not find any meaningful moderators for the intervention effect. Based on these findings, Macnamara and Burgoyne^13^ conclude that “apparent effects of growth mindset interventions on academic achievement are likely attributable to inadequate study design, reporting flaws, and bias” (p. 133). Commentaries by Yan & Schuetze^14^ and Tipton et al.^15^ identified a number of factors that might contribute to explaining these divergent findings, among others, differences in theoretical conceptualizations and measurement as well as differences regarding the applied meta-analytical approaches. In their commentary, Tipton et al.^15^ noted that the study by Burnette et al.^4^ was more in line with modern meta-analytical techniques for large, heterogeneous samples. Interestingly, when applying similar methods as considered in Burnette et al.^4^ to the dataset of Macnamara and Burgoyne^13^, Tipton et al.^15^ found comparable results to Burnette et al.^4^ that indicated a small positive effect of mindset interventions on student achievement. However, this interpretation has been contested^16^. Macnamara and Burgoyne, in their reply, argued that Tipton et al. made methodological errors in their re-analysis, including incorrect coding of studies and miscalculation of effect sizes. They asserted that when these errors were corrected, the re-analysis yielded results consistent with their original findings^9^, suggesting a negligible effect of mindset interventions on academic achievement.

One of the most prominent experimental studies was conducted by Yeager et al. ^3^ and published in *Nature*. In this study, 25 authors report on a growth mindset intervention of <1 h that substantially improved grades of lower-achieving students and enrollment in advanced math courses in a representative US sample of secondary school students. The authors found a positive average effect of 0.10 grade points on grade point averages (GPAs) and an increase of 3 percentage points in the likelihood of taking advanced mathematics courses in tenth grade. These positive results are largely in line with other studies such as one by Paunesku et al.^2^, who found an increase in GPAs of students who were poor performing and at risk of dropping out of high school. Other experimental studies contradict these findings. For instance, Li and Bates^17^ were not able to find growth mindset intervention effects on grades in a sample of Chinese students. The authors also noted that they did not detect any benefits of growth mindset interventions on students’ school attainment or their responses to failure. These results on missing main effects of growth mindset interventions on achievement are similar to other studies such as Burnette et al.^4^.

Observational studies have also typically found positive associations between students’ growth mindsets and their educational achievement. For instance, in a recent, high-quality observational study, Claro & Loeb^18^ investigated associations between growth mindset and student achievement in mathematics and English for a sample of >200,000 Californian students who were repeatedly assessed from grades 4 to 7. Using different regression models, the authors found that growth mindset was associated with an increase in English test scores of 0.07 SDs (about 18% of the annual growth) and an increase in math test scores of 0.04 SDs (about 17% of the annual growth). Importantly, as underscored by the authors, the study design and analyses do not allow results to be interpreted as causal estimates of growth mindset on student achievement.

Of particular relevance to our approach are observational studies that focused on the extent to which growth mindset mediates or moderates the effect of socioeconomic family background on educational achievement. In one highly influential study, Claro et al.^5^ found that Chilean students with a growth mindset were “appreciably buffered against the deleterious effects of poverty on achievement” (p. 8667). Closer scrutiny suggests that this claim is based on a comparison of low-income students with a growth mindset to high-performing students with a fixed mindset—a somewhat misleading comparison. More generally, the authors found that students from low-income families were less likely to hold a growth mindset, but that mindset was more important to their achievement. Based on these findings, they argue that mindsets might be an important mechanism through which economic disadvantages can affect achievement. However, they did not quantify the share of achievement inequality that is mediated through growth mindset. Destin et al.^19^ considered a nationally representative sample of 4,828 ninth-grade students from the United States to investigate this question. Using mediation analysis, the authors found that growth mindset explained only about 2% of the socioeconomic achievement gap. In another more recent observational study, Gruijters et al.^20^ found that, in sum, socioemotional skills (i.e., self-efficacy, competitiveness, fear of failure, work mastery, and growth mindset) can explain at most 8.8% of the achievement gap between low- and high-SES students. It is important to note that other studies using PISA data have also explored the role of growth mindset in achievement across multiple countries, though with varying findings. For example, Bernardo et al.^6^, using a multilevel approach with PISA 2018 data from 39 countries/territories, investigated how society-level social axioms influence the relationship between growth mindset and achievement across cultures. They reported significant variability in growth mindset effects across countries, finding weaker effects in societies with stronger social complexity beliefs. Similarly, Kaya et al.^7^ utilized PISA 2018 data to examine the relationships between growth mindset, resilience, and science achievement among immigrant students in Australia, the UK, and the USA. Their study found that growth mindset had a significant effect on science achievement, but the mediating role of attitudes toward school was not confirmed, and they speculated that self-theories might affect immigrant groups differently in different countries.

In sum, there are conflicting reports about the relationship between growth mindset and achievement inequality, partially because of different samples and analytical methods employed in previous studies.

## Results

### Main findings

We first examined and described the association between socioeconomic status, growth mindset, and student performance in the math, science, and reading sections of the PISA test. Following this, we conducted a 4-way decomposition analysis for the pool of countries, controlling for gender, immigration status, and country-fixed effects in each of the three subject areas: math, science, and reading. We focus here on the results for mathematics, as it was the primary domain in PISA 2022, and the patterns of student achievement across the three subjects were similar. However, the results for science and reading can be found in the Supplementary Information (Supplementary Figs. 2–4 and Supplementary Tables 1 and 2). As a robustness check, we also present in the Supplementary Information the results of models run without control variables for math, science, and reading (Supplementary Tables 3, 4, and 5), noting that the overall pattern of results is similar to those reported in the main text.

**Fig. 1:**
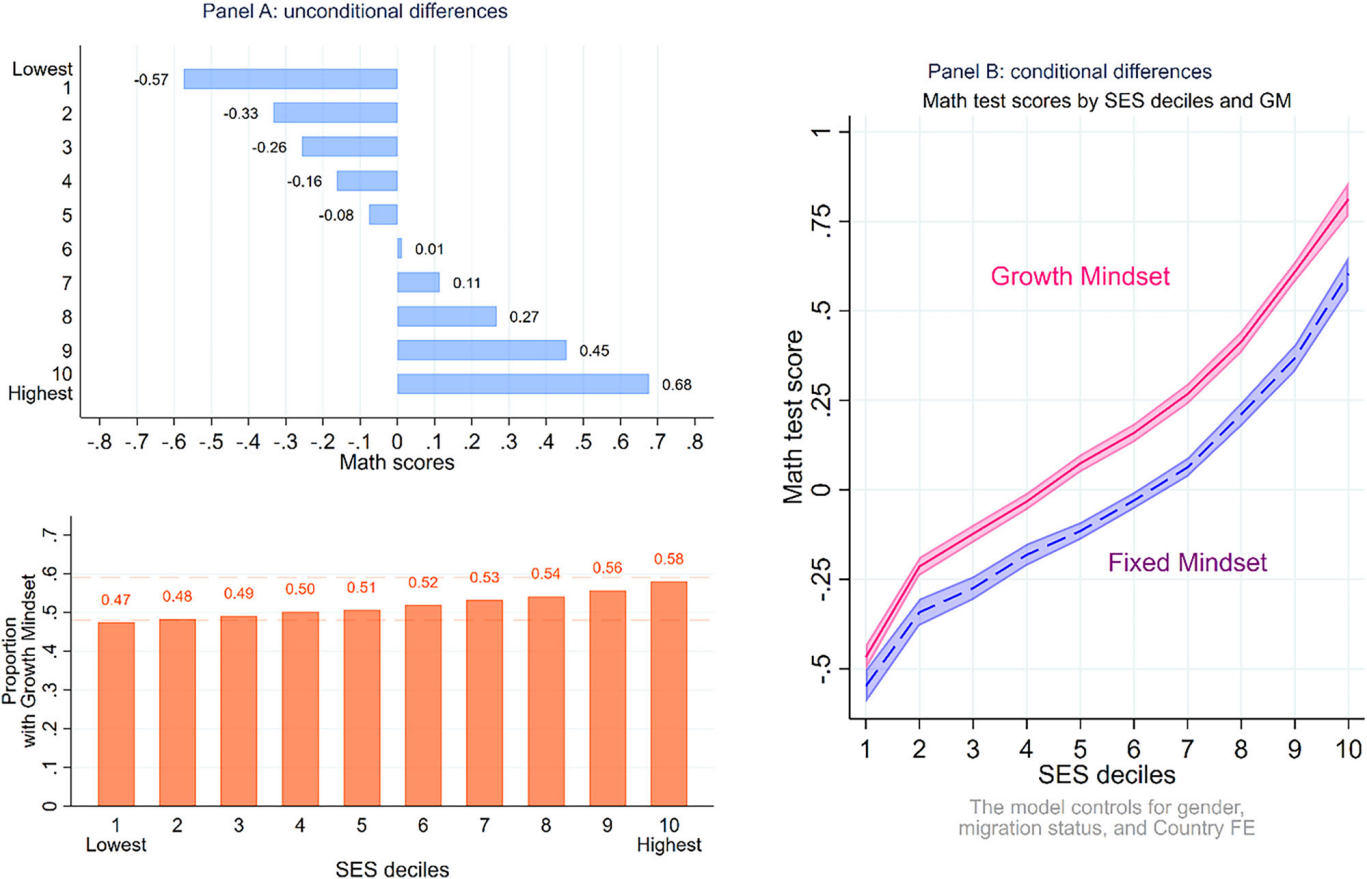
Association between PISA Test scores, SES, and GM. Figure 1 shows, in (Panel **A**), the distribution of Math scores across SES deciles, while (Panel **B**) illustrates the relationships between PISA Math scores, SES, and growth mindset across 73 countries. Higher SES is associated with better scores and a greater prevalence of growth mindsets. While growth mindset is linked to improved performance, higher SES consistently leads to better outcomes, particularly in Mathematics.

Figure 1 illustrates the association between PISA test scores, socioeconomic status (SES), and growth mindset (Panel a), averaged across the 73 countries. The plots show a clear positive relationship between SES and math test scores. Furthermore, the proportion of students with a growth mindset increases with higher SES levels, suggesting that students from more advantaged backgrounds are more likely to hold a growth mindset. Next, we conducted a linear regression analysis to examine the effects of having a growth mindset on math test scores across different socioeconomic status (SES) deciles, while controlling for gender, migration status, and country (Panel b). The results indicated that students who held a growth mindset demonstrated superior performance across all subjects compared to those with a fixed mindset. However, it is noteworthy that as SES levels increased, we consistently observed higher levels of test performance, irrespective of mindset. This suggests that while a growth mindset can benefit students across the socioeconomic spectrum, those from more advantaged backgrounds tended to achieve better outcomes overall. It also appears that growth mindset is less beneficial for learning at low levels of SES, contrary to much of the previous literature^5^.

We move next into the decomposition of the effect of socioeconomic status on the math test scores. As previously discussed, the four-way decomposition provides a unified approach to disentangle mediation and interaction mechanisms. It decomposes the total effect into four components with precise counterfactual definitions. This allows estimating not only the portion eliminated by fixing the mediator at a certain level (the CDE), but also the separate contributions due to mediation alone (the PIE), interaction alone in the presence of the mediator (the reference interaction), and the combined effect capturing both mediation and interaction mechanisms (the mediated interaction). Decomposing the total effect this way offers novel insights into how the mediation and interaction components interconnect to produce the observed effect size.

Table 1 present the main results from the four-way decomposition analysis for math averaged across the 73 countries, while Supplementary Tables 1 and 2 in the Supplementary Information section present the respective result for reading and science. The upper portion of each table displays the main effects, while the lower part shows the relative magnitude or importance of each of these effects. The first column illustrates the results when fully closing the sample gap in growth mindset between low- and high-SES students (0.55) in math. The subsequent columns then demonstrate how these results could change by fixing the mediator at different levels: 0, 0.2, 0.4, and 0.6. This allows for an examination of how varying the mediator values impacts the mediation effects on the student achievements across the different scenarios. To enhance clarity, we describe the results in relation to each research question and estimands of interest defined in Table 2, Methods section.

**Table 1:** 4-Way decomposition results, 73 countries, math test scores, PISA 2022

Effects	Closing Gap	Fixing M = 0	Fixing M = 0.2	Fixing M = 0.4	Fixing M = 0.6
TE	-0.587***	-0.591***	-0.587***	-0.587***	-0.587***
	(0.003)	(0.003)	(0.003)	(0.003)	(0.003)
	[-0.592, -0.581]	[-0.596, -0.586]	[-0.592, -0.581]	[-0.592, -0.581]	[-0.592, -0.581]
CDE	-0.575***	-0.529***	-0.543***	-0.561***	-0.579***
	(0.003)	(0.004)	(0.003)	(0.003)	(0.003)
	[-0.580, -0.570]	[-0.536, -0.521]	[-0.549, -0.537]	[-0.566, -0.556]	[-0.584, -0.574]
INT_ref	-0.000***	-0.051***	-0.032***	-0.014***	0.004***
	(0.000)	(0.003)	(0.002)	(0.001)	(0.000)
	[-0.001, -0.000]	[-0.057, -0.046]	[-0.036, -0.029]	[-0.016, -0.013]	[0.003, 0.004]
INT_med	0.006***	0.006***	0.006***	0.006***	0.006***
	(0.000)	(0.000)	(0.000)	(0.000)	(0.000)
	[0.005, 0.006]	[0.005, 0.006]	[0.005, 0.006]	[0.005, 0.006]	[0.005, 0.006]
PIE	-0.017***	-0.017***	-0.017***	-0.017***	-0.017***
	(0.000)	(0.000)	(0.000)	(0.000)	(0.000)
	[-0.018, -0.016]	[-0.017, -0.016]	[-0.018, -0.016]	[-0.018, -0.016]	[-0.018, -0.016]
Prop. CDE	0.980***	0.895***	0.926***	0.956***	0.987***
	(0.001)	(0.005)	(0.003)	(0.001)	(0.001)
	[0.979, 0.982]	[0.885, 0.904]	[0.920, 0.932]	[0.954, 0.959]	[0.985, 0.989]
Prop. INT_ref	0.001***	0.087***	0.055***	0.024***	-0.006***
	(0.000)	(0.005)	(0.003)	(0.001)	(0.000)
	[0.000, 0.001]	[0.077, 0.097]	[0.049, 0.062]	[0.022, 0.027]	[-0.007, -0.005]
Prop. INT_med	-0.010***	-0.010***	-0.010***	-0.010***	-0.010***
	(0.001)	(0.001)	(0.001)	(0.001)	(0.001)
	[-0.011, -0.008]	[-0.011, -0.008]	[-0.011, -0.008]	[-0.011, -0.008]	[-0.011, -0.008]
Prop. PIE	0.029***	0.028***	0.029***	0.029***	0.029***
	(0.001)	(0.001)	(0.001)	(0.001)	(0.001)
	[0.027, 0.030]	[0.027, 0.030]	[0.027, 0.030]	[0.027, 0.030]	[0.027, 0.030]
OP med	0.019***	0.018***	0.019***	0.019***	0.019***
	(0.001)	(0.001)	(0.001)	(0.001)	(0.001)
	[0.018, 0.020]	[0.017, 0.020]	[0.018, 0.020]	[0.018, 0.020]	[0.018, 0.020]
OP ati	-0.009***	0.077***	0.045***	0.015***	-0.016***
	(0.001)	(0.005)	(0.003)	(0.001)	(0.001)
	[-0.010, -0.008]	[0.069, 0.086]	[0.040, 0.051]	[0.013, 0.017]	[-0.018, -0.014]
OP eliminated	0.020***	0.105***	0.074***	0.044***	0.013***
	(0.001)	(0.005)	(0.003)	(0.001)	(0.001)
	[0.018, 0.021]	[0.096, 0.115]	[0.068, 0.080]	[0.041, 0.046]	[0.011, 0.015]
**Observations**	**503,656**	**503,656**	**503,656**	**503,656**	**503,656**

Standard errors in parenthesis; 95% Confidence intervals in brackets; *p < 0.10, **p < 0.05, ***p < 0.01. *TE* total effect, *CDE* controlled direct effect, *INT_ref* reference interaction, *INT_med* mediated interaction, *PIE* pure indirect effect, *Prop. CDE* proportion controlled direct effect, *Prop. INT_ref* proportion reference interaction, *Prop. INT_med* proportion mediated interaction, *prop. PIE* proportion pure indirect effect, *OP_med* overall proportion mediated, *OP_ati* overall proportion attributable to interaction, *OP_eliminated* overall proportion eliminated.

Regarding the overall difference in PISA learning scores between students with low versus high socioeconomic status (Total Effect, Q1 in Table 2), we find a substantial and highly significant gap. Students from lower SES backgrounds score 0.587 standard deviations lower in mathematics compared to their higher SES peers (*p* < 0.01). This total effect encompasses both direct influences of SES and those potentially mediated through growth mindset.

When examining what the SES achievement gap would be if all students had equal levels of growth mindset (CDE, Q2 in Table 2), we find that fixing growth mindset at different levels (0 to 0.6) yields gaps ranging from -0.529 to -0.579 standard deviations. Notably, the SES achievement gap actually slightly widens as the level of growth mindset increases, suggesting that equalizing mindset across all students would not reduce socioeconomic disparities in achievement.

The Reference Interaction (INT_ref, Q3 in Table 2), which captures how SES and mindset jointly affect achievement when SES does not influence mindset formation, shows minimal effects. When closing the sample gap in growth mindset, this interaction is nearly zero (-0.000), though it varies from -0.051 to 0.004 when fixing mindset at different levels. This suggests that the combination of SES and mindset levels has little impact on achievement beyond their separate effects.

The Mediated Interaction (Q4 in Table 2), representing how SES and mindset interact when SES influences mindset formation, shows a small but positive effect (0.006). This indicates that growth mindset slightly moderates the negative effect of low SES on achievement, but this moderating effect remains minimal, accounting for only -1% of the total effect (Prop. INT_med = -0.010).

Finally, examining how much of the SES achievement gap operates through mindset differences (PIE, Q5 in Table 2), we find a significant but small effect of -0.017 (*p* < 0.01). This means that SES-driven differences in mindset explain only 2.9% of the total achievement gap (Prop. PIE = 0.029), indicating that growth mindset plays a very limited mediating role in the relationship between SES and academic achievement.

The overall proportion mediated (OP_med = 0.019) and the proportion attributable to interaction (OP_ati = -0.009) further reinforce that while growth mindset does play a role in the SES-achievement relationship, its contribution is modest. The majority of the SES effect on mathematics achievement operates through direct pathways (Prop. CDE = 0.980) rather than through growth mindset or its interactions with SES.

This pattern of results is consistent across different specifications where we fix the mediator at various levels (0, 0.2, 0.4, and 0.6). While the magnitude of some effects varies across these specifications, the overall conclusion remains unchanged: growth mindset plays a relatively minor role in explaining or mitigating socioeconomic disparities in academic achievement.

The patterns observed in mathematics are largely consistent across science and reading (Supplementary Tables 1, and 2 in the Supplementary Information), though with some notable variations in effect magnitudes. The total effect of SES is strongest in mathematics (-0.587), followed by science (-0.543), and slightly lower in reading (-0.525), suggesting that socioeconomic background may have a somewhat stronger influence on mathematical skills. When examining the mediating role of growth mindset, we find similar modest effects across all three domains: the pure indirect effect (PIE) accounts for ~2.9% (CI 95%: 2.7, 3.0) of the total effect in mathematics, 3.2% (CI 95%: 3.1%, 3.4%) in science, and 3.2% (CI at 95%: 3.0%, 3.4%) in reading. The controlled direct effects (CDE) remain dominant across all subjects, accounting for 98% of the total effect in mathematics, 97.7% in science, and 97.8% in reading when closing the sample gap in growth mindset. The mediated interaction effects are consistently small and positive across all subjects (around 0.006), indicating that while growth mindset slightly moderates the negative effect of low SES on achievement, this moderating effect is minimal regardless of the subject area. These consistent patterns across different academic domains strengthen our main finding that while growth mindset may have some beneficial effects on academic achievement, its role in mediating or moderating socioeconomic disparities in educational outcomes is limited, regardless of the subject matter being assessed.

**Fig. 2:**
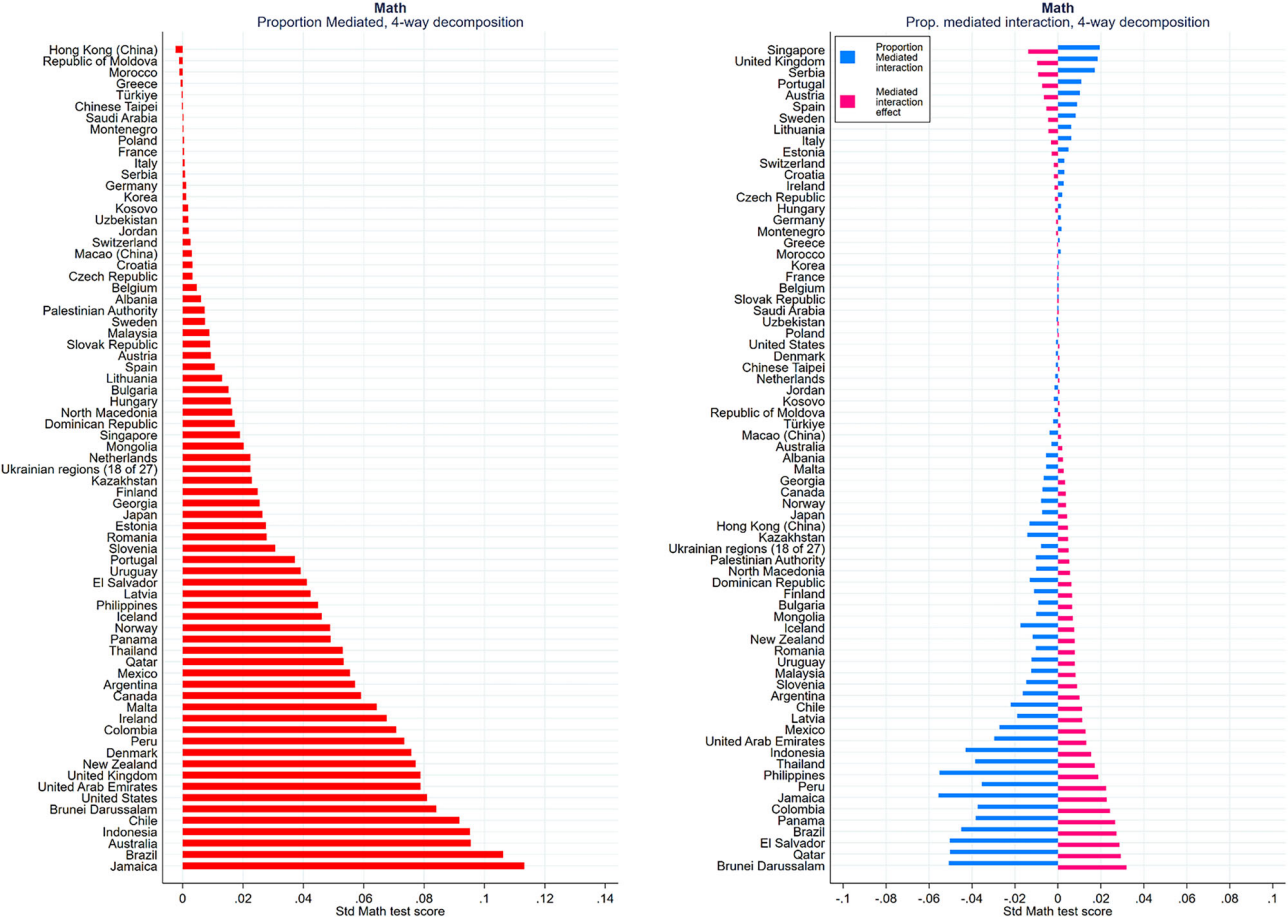
Proportion mediated (PIE) in 4-Way decomposition by country. Figure 2 shows cross-national variation in growth mindset’s mediation and moderation of SES’s effect on math scores. Mediation is small (0 - 0.11%). Positive moderated interaction (up to 0.038) indicates slight mitigation of low SES impact, but overall effect is limited.

Until now, we have looked at averages across countries. It is likely, however, that the interrelationships between socioeconomic family background, growth mindset, and academic achievement vary across countries and education systems. Thus, Fig. 2 left panel, presents the proportion mediated (PIE) for each country in math test score, ordered from smallest to largest, highlighting the cross-national variability in the extent to which growth mindset mediates the SES effect on learning outcomes. While a few countries exhibit higher proportions mediated — such as Chile, Indonesia, Australia, Brazil, and Jamaica, where the proportion mediated reaches a maximum of 0.11 — the majority of countries fall within the range of 0 to 0.08, reinforcing the overall finding that growth mindset plays a relatively minor role in explaining socioeconomic disparities in academic achievement across different contexts.

Finally, Fig. 2, right panel, also presents the proportion mediated interaction and the mediated interaction effect (INT_med, pink bars) for math test score across various countries, with countries ranked smallest to largest values (as before, science, and reading test scores available in the Supplementary Information). The mediated interaction effect (INT_med) captures how the influence of low SES on learning outcomes is modified by growth mindset. A positive interaction effect indicates that for students with a growth mindset, the detrimental effect of low SES on test scores is less severe than it would be for students with a fixed mindset. In other words, growth mindset mitigates some of the negative impact of low SES, thus reducing the SES-based achievement gap. This positive interaction effect is observable across multiple countries and test subjects (math, science, and reading), though the magnitude of this effect remains small overall. Countries such as Colombia, Panama, Brazil, El Salvador, Qatar, and Brunei in math exhibit stronger positive mediated interaction effects (max.038), indicating a more pronounced role for growth mindset in narrowing the SES gap in these regions.

Conversely, the proportion mediated interaction (blue bars) quantifies the extent to which the mediated interaction effect contributes to the overall effect of SES on test scores. Despite the positive mediated interaction effect (pink bars), the proportion of this interaction is negative in most countries. This occurs because, while the INT_med is positive, the total effect of SES on learning outcomes is negative. The negative proportion suggests that while the beneficial role of growth mindset counteracts the broader negative effects of low SES on test performance, its moderating effect is small and does not fully overcome these disadvantages. For example, the largest values are around -0.06 for Jamaica and Philippines, followed by -0.05 for Qatar and Brunei.

In summary, our results indicate a strong direct effect of SES on PISA test scores, with growth mindset mediating only a small proportion of this effect, averaging around 2.9% for math and 3.2% science and reading. The findings challenge prior studies notion that growth mindset interventions could serve as a powerful tool for policy makers to mitigate educational inequalities across diverse socioeconomic contexts.

Additionally, to contextualize these findings, we consider three important limitations of the study. First, the estimates of mediation effects presented in this study should be interpreted as upper bounds of the true causal effect. This is because the analyses assume that the relationships between SES, growth mindset, and achievement are unconfounded and that no reverse causality is present. These assumptions may not fully hold in the observational data used. One key limitation is the potential for reverse causality, whereby academic achievement influences growth mindset, rather than the reverse. For example, students who perform well academically may internalize the belief that their success stems from effort and improvement, thereby reinforcing a growth mindset. If this reverse pathway exists, it could inflate the observed mediation effect, making it appear as though growth mindset has a larger impact on achievement than it actually does.

Furthermore, unmeasured confounders may also introduce upward bias in the estimated effects. For instance, variables such as parental attitudes, teacher expectations, school resources, or peer influences could independently affect both growth mindset and academic performance. These factors are often correlated with SES and could introduce upward bias if they create the appearance of a stronger mediating role for growth mindset than exists in reality. Violations of the sequential ignorability assumption —particularly for mediator-outcome confounding could lead to upward bias in the estimated mediation effect if unmeasured factors that are correlated with both the mediator (growth mindset) and the outcome (academic achievement) are omitted from the analysis. For instance, if teacher support or school quality simultaneously enhances growth mindset and academic outcomes, these omitted variables could create the illusion of a stronger mediating role for growth mindset than actually exists. Similarly, if parental involvement, which tends to correlate with SES (another unobserved mediated path), also fosters both a growth mindset and better academic performance, failing to account for this factor would inflate the apparent effect of growth mindset on achievement. Such violations could create the illusion that growth mindset accounts for a larger portion of the SES effect on achievement than the data actually support.

Finally, this study is limited by its use of PISA scores to measure academic achievement. Although PISA offers a standardized metric for cross-national analysis, it may not reflect the full range of skills and abilities that growth mindset promotes. Different countries employ varied assessment approaches, which may emphasize different learning outcomes, potentially influencing the observed relationship with growth mindset. Importantly, despite these potential measurement limitations, our analyses consistently revealed small mediated effects of growth mindset across countries.

Taken together, these limitations suggest caution in interpreting the modest mediation effects as evidence of a substantial causal role for growth mindset in mitigating SES-based disparities in academic achievement and, at best, suggest a conservative and small effect of growth mindset. While growth mindset interventions may still provide meaningful benefits for individual students, their capacity to reduce systemic educational inequalities appears limited. Future research using longitudinal and panel designs to track students’ growth mindsets, educational progress, and performance from early stages would be critical for addressing these limitations and disentangling the complex causal pathways between SES, growth mindset, and academic achievement.

## Discussion

The widespread promotion of growth mindset as a potential solution to socioeconomic disparities in education has been underpinned by studies claiming it is particularly beneficial for students from disadvantaged backgrounds. However, closer examination of this body of work reveals substantial methodological shortcomings, including overreliance on single-country samples, unclear definitions of effects and estimands of interest, and a tendency to overstate the role of mindset interventions in mitigating structural disadvantages. Additionally, while a limited number of cross-country or comparative studies have explored the role of growth mindset in achievement, these studies have generally not focused on the mediating role of growth mindset in the relationship between socioeconomic status and academic achievement across diverse national contexts. Our study directly addresses these limitations by utilizing a more advanced decomposition approach, applied to data from 73 countries in the 2022 PISA assessment. This comprehensive analysis allows us to isolate and quantify the relative importance of direct, indirect, and interaction effects of socioeconomic status (SES) and growth mindset on academic outcomes. In contrast to earlier claims, our findings demonstrate that while growth mindset has a modest positive association with achievement, its capacity to meaningfully reduce SES-based inequalities is limited.

Importantly, unlike earlier studies that relied on regression-based approaches, our four-way decomposition precisely delineates the direct effect of SES, the indirect effect operating through growth mindset, and potential interaction effects. This analytical clarity addresses concerns about overcontrolling bias and unclear effect comparisons that have plagued prior work.

Overall, we found little evidence in support of the hypothesis that a growth mindset is crucial for reducing socioeconomic disparities in learning outcomes. Across the 73 countries analyzed, the direct effect of SES on PISA test scores was consistently large and highly significant, even after accounting for potential mediation by growth mindset. Conversely, the indirect effect mediated through growth mindset was comparatively small, accounting on average for only 2.9% (CI at 95%: 2.7%, 3.0%) of the total SES effect on math achievement, with similar results for science (3.2%; CI at 95%: 3.1%, 3.4%) and reading (3.2%; CI at 95%: 3.0%, 3.4%) achievements. This finding aligns with the results of Gruijters et al.^20^, who also found limited explanatory power of socioemotional skills in accounting for achievement gaps. While the mediated effect varied somewhat across countries, the overwhelming pattern indicated a modest role for growth mindset in explaining educational inequalities. This variability across countries, consistent with Bernardo et al.’s finding^6^ that the growth mindset-achievement relationship varies culturally, suggests that the influence and mediating role of growth mindset are context-dependent and potentially weaker in countries with less supportive cultural norms, even among disadvantaged students. Moreover, potential unmeasured confounders and reverse causality are likely to upwardly bias these results, making the true effect of growth mindset appear larger than it actually is, which further casts doubt on its significance in addressing educational inequalities. This finding, therefore, challenges the notion proposed by previous studies^4,5^ that growth mindset interventions could serve as a powerful policy tool for mitigating socioeconomic gaps in academic performance. Even if such interventions could fully close the gap in growth mindset between low- and high-SES students, which seems unlikely, the effect on the socioeconomic achievement gap would be minor. The four-way decomposition approach further revealed that interaction effects between SES and growth mindset were generally small and inconsistent in magnitude and direction. This suggests that the interplay between these two factors does not substantially alter the overall effects on student achievements. While growth mindset may still offer benefits for individual students’ motivation and engagement, our findings indicate that addressing systemic socioeconomic disparities in educational opportunities and resources remains a crucial priority for policymakers aiming to promote more equitable learning outcomes. More broadly, our results have several implications for both policy and future research. Firstly, the limited overall mediating effect of growth mindset on the SES-achievement link suggests that interventions solely focused on cultivating this individual-level trait may have a limited impact on reducing educational inequalities. Instead, greater emphasis and resources should likely be directed towards addressing systemic factors such as school resources, teacher quality, and parental involvement, which our study implies may play a more substantial role. Secondly, the significant cross-national variability in the mediating effect underscores the importance of cultural context. Educational policies and interventions related to growth mindset should be carefully adapted to align with specific cultural norms and values, as a ‘one-size-fits-all’ approach is unlikely to be effective. Finally, future research should prioritize exploring the complex interplay between individual psychological constructs like growth mindset and broader macro-level societal and cultural influences to gain a more nuanced understanding of achievement gaps and identify more effective levers for change.

## Methods

### Data

We used data from the latest Programme for International Student Assessment (PISA), conducted in 2022 by the Organisation for Economic Co-operation and Development (OECD)^12^. The PISA dataset offers a unique opportunity to study variability across countries, overcoming a limitation of previous studies on growth mindset that focused on single-country analyses. With internationally comparable student assessment scores and extensive background information, PISA enables a comprehensive cross-country investigation of the relationship between growth mindset and learning outcomes. Our analysis included a sample of 73 countries after dropping several countries due to missing data. Specifically, we excluded Cambodia, Guatemala, Israel, Paraguay, Viet Nam, and Baku (Azerbaijan) as they did not assess growth mindset. We also dropped Costa Rica because the socioeconomic status (SES) variable was missing.

Our main outcome of interest was the first plausible value of PISA test scores in mathematics, which we standardized within each country to have a mean of 0 and a standard deviation of 1. Results for Science and Reading scores were substantively similar and are presented in the Supplementary Information. The key explanatory variable was growth mindset, an ordinal scale with four categories: i) Strongly disagree, ii) Disagree, iii) Agree, iv) Strongly agree. The question used to assess growth mindset was: “*To what extent do you agree or disagree with the following statement: Your intelligence is something about you that you cannot change very much*”. Following the literature^5^, we recoded this into a dichotomous variable that distinguishes between students with a growth mindset and those with a fixed mindset. The exposure variable was the PISA index of economic, social, and cultural status (ESCS), which served as a proxy for socioeconomic status. The ESCS index was derived from three variables related to family background: parents’ highest level of education (PARED), parents’ highest occupational status (HISEI), and home possessions (HOMEPOS), including the number of books in the home^21^. For descriptive statistics, we recoded the ESCS index into ten deciles, standardized within countries, and for the regression analysis, we created a dichotomous variable indicating whether the student was above or below the median ESCS value in their respective country. This dichotomization was chosen to optimize sample sizes when testing interaction effects. Additionally, we included the following exogenous covariates in our analysis: gender (boys and girls) and migration status (native, first-generation, second-generation, missing). Gender and migration status were included as covariates in our analysis due to their potential influence on student mindsets and academic performance. Previous research has documented gender differences in academic mindsets, with girls often exhibiting lower self-confidence and more fixed mindsets compared to boys, despite outperforming them in certain subjects^22,23^. Accounting for gender was therefore crucial to avoid confounding and to explore potential heterogeneous effects of growth mindset. Similarly, migration status can shape students’ educational experiences, resources, and mindsets. First- and second-generation immigrant students may face unique challenges, such as language barriers, cultural adjustments, and disparities in socioeconomic status, which could impact their academic mindsets and performance^24^. By controlling for migration status, we aimed to disentangle its effects from those of growth mindset and socioeconomic factors, providing a more nuanced understanding of the relationships under investigation. Finally, we applied senate weights to all descriptive figures and statistical models to ensure equal representation of each country in the analysis. All the analysis were performed using Stata version 17 and the package Med4way^25^.

**Table 2:** Definitions of the 4-Way decomposition as applied to the current study

Research Question	Component	Definition	Interpretation
Q1: What is the difference in PISA learning scores between students with low vs high socioeconomic status?	Total effect (TE)	Total effect of A (changing *a’* to *a*) on Y	This represents the overall effect of SES on achievement scores (math, science, or reading), encompassing both direct influences and those mediated by growth mindset.
Q2: What would the difference in PISA learning scores be between students with low vs high SES if all students had a growth mindset equal to *m*?	Controlled direct effect (CDE)	Effect of A (changing *a’* to *a*) on Y, intervening to fix M to *m*	This reflects the effect of SES on achievement scores when growth mindset is held constant, illustrating how SES influences achievement independently of mindset.
Q3: What is the combined difference in PISA learning scores between students with low SES and fixed mindset vs high SES and growth mindset if SES does not affect mindset?	Reference interaction (INT_ref)	An additive interaction that operates only if M is present when A is *a*	This captures how the effect of SES on achievement differs between students with a fixed mindset and those with a growth mindset, specifically at the reference level of mindset.
Q4: What is the combined difference in PISA learning scores between students with low SES and fixed mindset vs high SES and growth mindset if SES affects mindset?	Mediated interaction (INT_med)	An additive interaction that operates only if A (changing *a’* to *a*) has an effect on M	This represents how the interaction between SES and growth mindset operates through the mediation pathway, highlighting differences in the mediating effect of growth mindset across various SES levels.
Q5: What is the difference in PISA learning scores for students with fixed vs growth mindset if SES determines mindset?	Pure indirect effect (PIE)	Effect of the mediator (changing *m’* to *m*) on Y when A is _*a*_, multiplied by the effect of A (changing *a’* to *a*) on M	This effect operates solely through changes in growth mindset due to SES, indicating how SES influences achievement indirectly without interaction.

### Analytical Approach

The standard approach in the existing growth mindset literature has been to investigate the association between socioeconomic status (SES), growth mindset, and educational outcomes using a regression framework. In this approach, SES and growth mindset are included as concomitant predictors of the outcome variable, and in some cases, an interaction term between SES and growth mindset is added to understand how the effects of growth mindset might differ for students from low versus high SES backgrounds (see Fig. 1, panel a). The directed edges indicate effects of the SES and Growth Mindset on the outcome Y. The undirected dash line denotes a correlation between the two key explanatory variables instead.

**Fig. 3:**
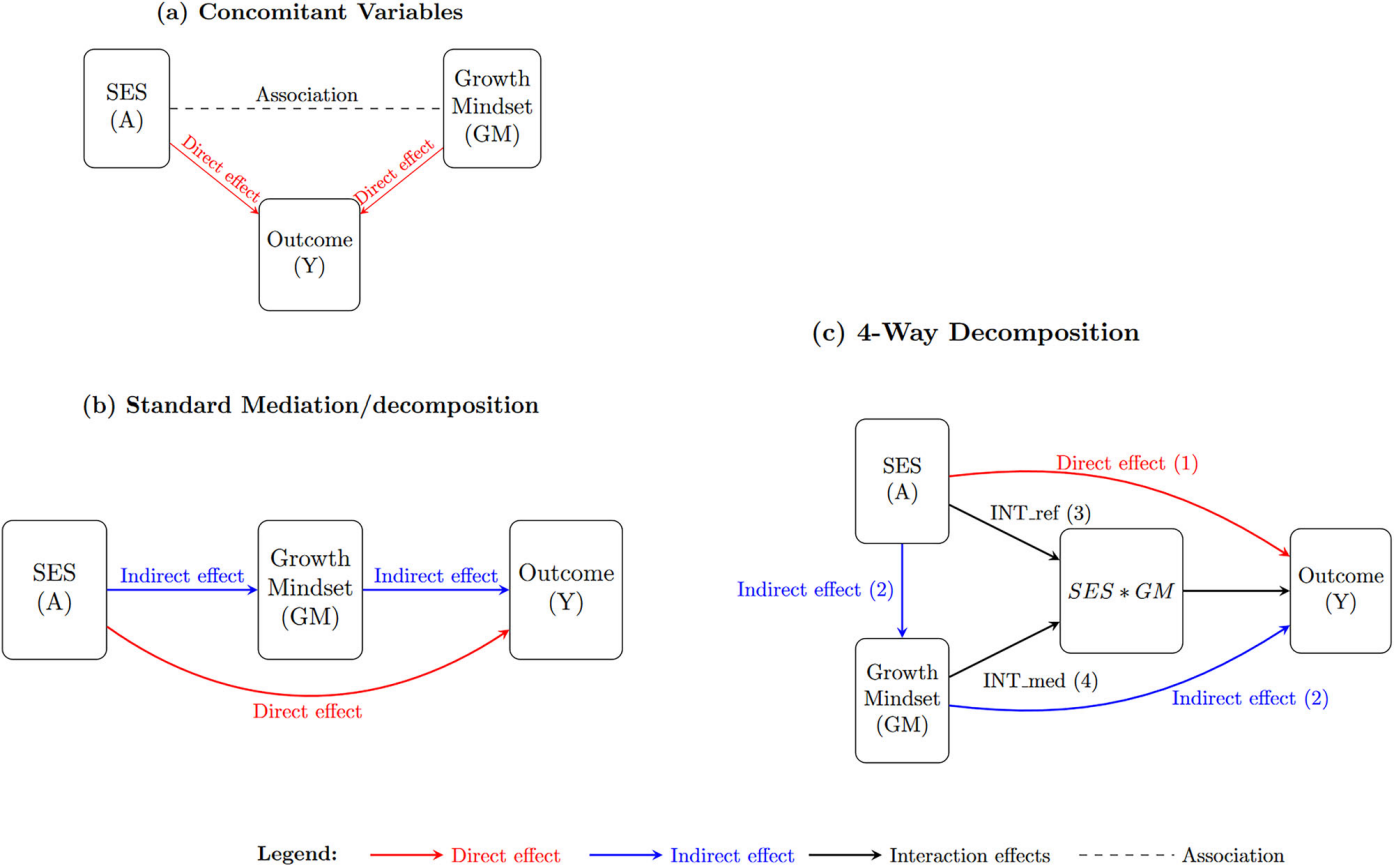
Causal diagram for conceptualizing the relationship between SES, GM and Student achievements. Figure 3 shows frameworks for analysing socioeconomic status (SES), growth mindset, and academic achievement. It includes regression (Panel **A**), standard mediation (Panel **B**), and a four-way decomposition (Panel **C**) to detail direct, indirect, and interaction effects, providing comprehensive insights. **Note:** Diagram in Panel c illustrates the 4-Way decomposition of the total effect of socioeconomic status (SES) on PISA learning scores (Y) via growth mindset (GM). The interaction between SES and growth mindset is captured by the term GM*SES. Arrow 1 represents the Controlled Direct Effect (CDE): the effect of SES (exposure) on PISA scores (outcome), independent of growth mindset (mediator). This shows how SES influences academic achievement when growth mindset is held constant. Arrow 2 represents the Pure Indirect Effect (PIE): the effect of SES on PISA scores solely through growth mindset, without any interaction between SES and growth mindset. Arrow 3 represents the Reference Interaction (INT_ref): the interaction between SES and growth mindset, reflecting how the effect of SES on achievement differs based on students’ mindset when SES has no effect on mindset. Arrow 4 represents the Mediated Interaction (INT_med): the interaction between SES and the component of growth mindset that is influenced by SES. This highlights how the interaction between SES and achievement operates through the mediation of growth mindset.

We argue that this approach can lead to several issues. First, growth mindset is likely to be endogenous to SES, as children from high-SES backgrounds may be more likely to develop a growth mindset due to their environment and experiences^5^. If this is the case, growth mindset acts as a mediator variable, as represented in Fig. 3, panel b, and including it alongside SES in the regression would block part of the effect of SES^26,27^. Second, comparing the (crude) magnitudes of the coefficients of SES and growth mindset could lead to misleading conclusions, as including the mediator variable (growth mindset) may underestimate the true effect of SES.

To address these limitations, we study the relationship between SES, growth mindset, and student achievements within a counterfactual framework, using a four-way decomposition approach^10,25^. This allows us to disentangle the direct and indirect effects of SES on student achievements, identifying growth mindset as a potential mediating pathway. By integrating the counterfactual framework with a mediation perspective, we can better understand the mechanisms through which SES influences students’ achievements and the role of growth mindset in this process. This approach is represented in Fig. 3, panel b and c in which we compare the standard and the four-way decompositions.

In this framework, the total effect of socioeconomic status (SES) on student achievements can be divided into a direct effect and an indirect effect mediated by growth mindset (panel b, standard mediation/decomposition). While this is a simplified representation, it clarifies the relationships among the variables and their temporal order, which has surprisingly been overlooked in the existing literature on growth mindset. We enhanced this mediation model by incorporating the interaction between SES and growth mindset, which reveals distinct pathways through which SES can influence the outcome, including direct effects, indirect effects via growth mindset, and two potential interaction effects which we further describe below (panel c, four-way decomposition).

By adopting a mediation approach within a potential outcome framework, we can provide a clearer and more comprehensive understanding of the relative importance of growth mindset in mitigating socioeconomic inequalities in education. In particular, this approach allows us to avoid the potential issues of overcontrolling bias and misleading comparisons of coefficient magnitudes that can arise from the standard regression-based approach previously employed in this field^24^.

More formally, we translate our diagram in Fig. 3, panel c, into testable estimands following a potential outcome framework for mediation analysis as suggested by the latest literature on mediation analysis^10,28,29^. Notably, we follow^10,11^ who proposed 4-Way Decomposition, which disentangles the Total Effect (TE) of SES on the learning outcome into four components based on mediation and interaction effects:

*Due neither to mediation nor interaction* = Controlled Direct Effect (CDE);

*Due to interaction only* = Reference Interaction (INT_ref)

*Due to mediation and interaction* = Mediated Interaction (INT_med)

*Due to mediation only* = Mediated main effect or Pure indirect effect (PIE)

The TE is the sum of all four components as shown in Eq. 1:

1 $${TE}={CDE}+{{INT}}_{\_{ref}}+{{INT}}_{\_{med}}+{PIE}$$

Defining a binary exposure A[0,1], a binary mediator M[0,1], and the outcome variable as Y, we can express TE and its components as (Eq. 2):

2 $$\begin{array}{ccc}{Y}_{1}-{Y}_{0} & = & \left({Y}_{10}-{Y}_{00}\right)\\ & & +\left({Y}_{11}-{Y}_{10}-{Y}_{01}+{Y}_{00}\right)\left({M}_{0}\right)\\ & & +\left({Y}_{11}-{Y}_{10}-{Y}_{01}+{Y}_{00}\right)\left({M}_{1}-{M}_{0}\right)\\ & & +\left({Y}_{01}-{Y}_{00}\right)\left({M}_{1}-{M}_{0}\right)\end{array}$$

Importantly, we can connect each of the four estimands in the decomposition to specific research questions of interest. Table 2 provides definitions and explanations for each component, as well as how they can be applied to the current study.

The total effect (TE) represents the overall difference in PISA learning scores between students with low versus high socioeconomic status (SES). This captures the total impact of SES on academic achievement.

The controlled direct effect (CDE) represents what the difference in PISA scores would be between low and high SES students if we intervened to fix growth mindset at a specific level (e.g., if all students had the same growth mindset). This allows us to isolate the direct effect of SES on learning, net of any indirect pathway through growth mindset.

The reference interaction (INT_ref) represents an additive interaction effect that would only operate if growth mindset is present when SES changes. This captures the combined difference in scores between low SES/fixed mindset and high SES/growth mindset students, assuming SES does not affect mindset.

The mediated interaction (INT_med) represents an additive interaction effect that would only operate if SES has an effect on growth mindset. This captures the combined difference in scores between low SES/fixed mindset and high SES/growth mindset students, accounting for the fact that SES may shape mindset.

Finally, the pure indirect effect (PIE) represents the difference in PISA scores that is attributable to the effect of SES on growth mindset, and the subsequent effect of growth mindset on learning. This isolates the indirect pathway through which SES influences achievement.

By decomposing the total effect in this way, we can provide a more nuanced and comprehensive understanding of the complex interplay between SES, growth mindset, and educational outcomes. This allows us to better evaluate the extent to which growth mindset may serve as a mechanism for mitigating socioeconomic inequalities in learning.

The estimation involves using a linear regression model for the continuous outcome variable Y and a logistic regression model for the binary mediator M to calculate the various bivariate associations and decomposition components. Specifically, the average controlled direct effect (CDE) and the average pure indirect effect (PIE) are calculated using the estimated regression coefficients, controlling for exogenous covariates such as migration status, gender, and country. The reference interaction (INT_ref) is the difference between the pure direct effect and the controlled direct effect, while the mediated interaction (INT_med) is the difference between the total indirect effect and the pure indirect effect. These expressions allow for the estimation of the total effect (TE) and its decomposition into the various components without requiring the estimation of the full set of potential outcomes. For these analyses, we employed the package developed by Discacciati et al.^25^, as the four-way decomposition provides a more nuanced understanding of mediation effects by explicitly accounting for interactions between the mediator and exposure. Standard mediation approaches often fail to disentangle these interactive effects, potentially conflating direct and indirect effects^10^. This lack of separation can obscure the underlying mechanisms, leading to incomplete or misleading interpretations of how SES influences learning outcomes through growth mindset. Full details of the estimation procedure are provided in the Supplementary information section.

## Supplementary information

Supplementary Information

## Data Availability

The data used in this study are publicly available from the OECD Programme for International Student Assessment (PISA) 2022 database, accessible upon registration at: https://www.oecd.org/en/data/datasets/pisa-2022-database.html.

## Code availability

The analysis code is available upon request from the corresponding author.

## References

[1] Dweck, C. S. *Mindset: The New Psychology of Success*, Vol. 320 (Ballantine Books, 2007).

[2] Paunesku, D. et al. Mind-set interventions are a scalable treatment for academic underachievement. *Psychol. Sci.* **26**, 784–793 (2015). 25862544 10.1177/0956797615571017

[3] Yeager, D. S. et al. A national experiment reveals where a growth mindset improves achievement. *Nature* **573**, 364–369 (2019). 31391586 10.1038/s41586-019-1466-y PMC6786290

[4] Burnette, J. L. et al. A systematic review and meta-analysis of growth mindset interventions: for whom, how, and why might such interventions work?. *Psychol. Bull.* **149**, 174–205 (2023). 36227318 10.1037/bul0000368

[5] Claro, S., Paunesku, D. & Dweck, C. S. Growth mindset tempers the effects of poverty on academic achievement. *Proc. Natl. Acad. Sci. USA* **113**, 8664–8668 (2016). 27432947 10.1073/pnas.1608207113 PMC4978255

[6] Bernardo, A. B. I., Cai, Y. & King, R. B. Society-level social axiom moderates the association between growth mindset and achievement across cultures. *Br. J. Educ. Psychol.* **91**, 1166–1184 (2021). 33576017 10.1111/bjep.12411

[7] Kaya, S., Eryilmaz, N. & Yuksel, D. The effects of growth mindset and resilience on immigrant students’ PISA science achievement: the mediating role of attitudes toward school. *SAGE Open* **14**, 1–15 (2024).

[8] Lundberg, I., Johnson, R. & Stewart, B. M. What is your estimand? defining the target quantity connects statistical evidence to theory. *Am. Sociol. Rev.* **86**, 532–565 (2021).

[9] Rubin, D. B. Causal inference using potential outcomes: Design, modeling, decisions. *J. Am. Stat. Assoc.* **100**, 322–331 (2005).

[10] VanderWeele, T. J. A unification of mediation and interaction: a 4-way decomposition. *Epidemiology* **25**, 749–761 (2014). 25000145 10.1097/EDE.0000000000000121 PMC4220271

[11] Valeri, L. & VanderWeele, T. J. Mediation analysis allowing for exposure–mediator interactions and causal interpretation. *Psychol. Methods* **18**, 137–150 (2013). 23379553 10.1037/a0031034 PMC3659198

[12] OECD. *PISA 2022 Database* https://www.oecd.org/pisa/data/2022database/ (2022).

[13] Macnamara, B. N. & Burgoyne, A. P. Do growth mindset interventions impact students’ academic achievement? a systematic review and meta-analysis with recommendations for best practices. *Psychol Bull.* **149**, 133–173 (2023). 36326645 10.1037/bul0000352

[14] Yan, V. X. & Schuetze, B. A. What is meant by ‘growth mindset’?. *Psychol. Bull.* **149**, 206–219 (2023).

[15] Tipton, E. et al. Why meta-analyses of growth mindset and other interventions should follow best practices. *Psychol. Bull.* **149**, 229–241 (2023). 37701627 10.1037/bul0000384 PMC10495100

[16] Macnamara, B. N. & Burgoyne, A. P. A spotlight on bias in the growth mindset intervention literature: a reply to commentaries that contextualize the discussion and illustrate the conclusion. *Psychol. Bull.* **149**, 242–258 (2023).

[17] Li, Y. & Bates, T. C. You can’t change your basic ability, but you work at things. *J. Exp. Psychol. Gen.* **148**, 1640–1655 (2019). 31464486 10.1037/xge0000669

[18] Claro, S. & Loeb, S. Students with growth mindset learn more in school. *Educ. Res.* **53**, 389–402 (2024).

[19] Destin, M., Hanselman, P., Buontempo, J., Tipton, E. & Yeager, D. S. Do student mindsets differ by socioeconomic status?. *AERA Open* **5**, 1–12 (2019). 10.1177/2332858419857706 PMC7156083 32292799

[20] Gruijters, R. J., Raabe, I. J. & Hübner, N. Socio-emotional skills and the socioeconomic achievement gap. *Sociol. Educ.* **97**, 120–147 (2023).

[21] OECD. *PISA 2022 Results (Volume II): Learning During – and From – Disruption.* https://www.oecd.org/en/publications/pisa-2022-results-volume-ii_a97db61c-en.html (2022).

[22] Parker, P. D., Van Zanden, B. & Parker, R. B. Girls get smart, boys get smug. *Learn. Instr.* **54**, 125–137 (2018).

[23] Zander, L. et al. When grades are high but self-efficacy is low. *Front. Psychol.* **11**, 552355 (2020). 33162905 10.3389/fpsyg.2020.552355 PMC7580255

[24] Chesters, J. Does migrant status affect educational achievement, aspirations, and attainment? *Multicult*. *Educ. Rev.* **7**, 197–212 (2015).

[25] Discacciati, A., Bellavia, A., Lee, J. J., Mazumdar, M. & Valeri, L. Med4way: A Stata command to investigate mediating and interactive mechanisms using the four-way effect decomposition. *Int. J. Epidemiol.* **47**, 15–25 (2018). 30452641 10.1093/ije/dyy236

[26] Elwert, F. & Winship, C. Endogenous selection bias: the problem of conditioning on a collider variable. *Annu. Rev. Sociol.* **40**, 31–53 (2014). 30111904 10.1146/annurev-soc-071913-043455 PMC6089543

[27] VanderWeele, T. J., Mathur, M. B. & Chen, Y. Outcome-wide longitudinal designs for causal inference: a new template for empirical studies. *Stat. Sci.* **35**, 437–466 (2020).

[28] Pearl, J. The causal mediation formula—A guide to the assessment of pathways and mechanisms. *Prev. Sci.* **13**, 426–436 (2012). 22419385 10.1007/s11121-011-0270-1

[29] VanderWeele, T. J. Mediation analysis: a practitioner’s guide. *Annu. Rev. Public Health* **37**, 17–32 (2016). 26653405 10.1146/annurev-publhealth-032315-021402

