# Supplementary material for: Growth mindset and socioeconomic inequality in academic achievement across seventy-three PISA countries
Source: NPJ Sci Learn. 2025 Nov 20;10:81. doi: 10.1038/s41539-025-00365-8 (PMC12635329; doi:10.1038/s41539-025-00365-8)
Supplement: Supplementary file 1 — Supplementary Information [file 41539_2025_365_MOESM1_ESM.pdf]

Supplementary Information

A. Further detail on data and sample description

Supplementary Figure 1: Countries with more than 10% of missingness in growth mindset variable

Supplementary Figure 1 shows the percentage of missing data for growth mindset variables in Math and Intelligence across various countries from PISA 2022.

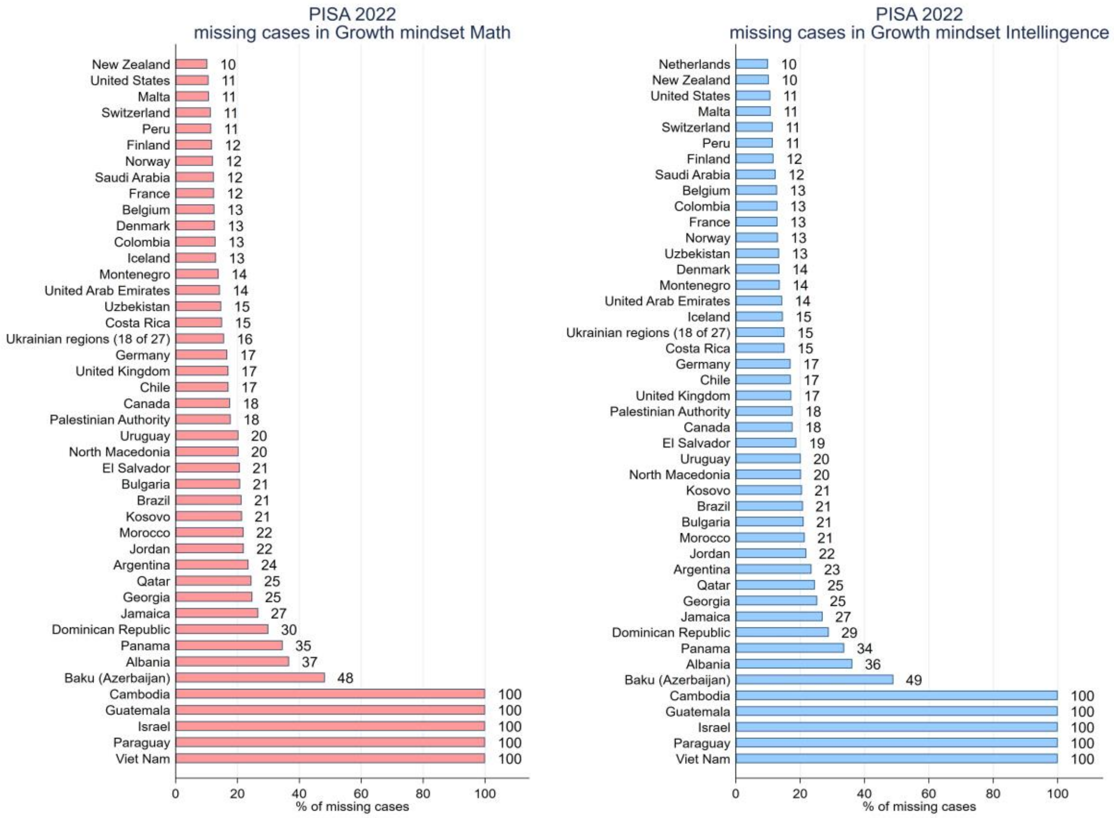

B. Additional results

Supplementary 2: Descriptive stats for math, science and reading test scores compared

Supplementary Figure 2 shows descriptive statistics for PISA math, reading, and science test scores. Panel A displays unconditional differences, while Panel B presents conditional differences by SES deciles and Growth Mindset.

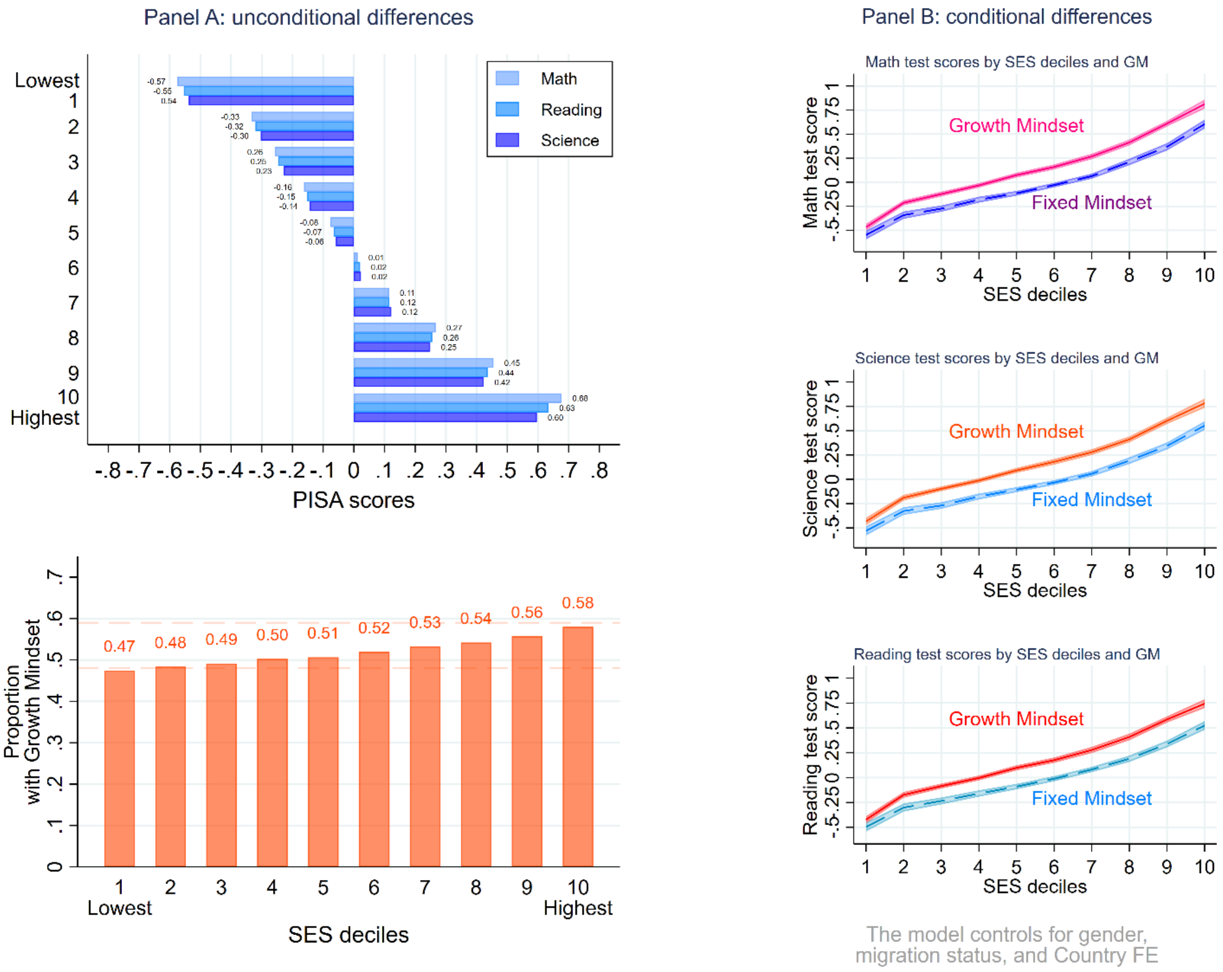

Supplementary Table 1: 4-Way decomposition results, 73 countries, science test scores, PISA 2022

Effects	Closing Gap	Fixing M = 0	Fixing M = 0.2	Fixing M = 0.4	Fixing M = 0.6
TE	-0.552***	-0.552***	-0.552***	-0.552***	-0.552***
	(0.003)	(0.003)	(0.003)	(0.003)	(0.003)
	[-0.557,-0.546]	[-0.557,-0.546]	[-0.557,-0.546]	[-0.557,-0.546]	[-0.557,-0.546]
CDE	-0.539***	-0.490***	-0.508***	-0.525***	-0.543***
	(0.003)	(0.004)	(0.003)	(0.003)	(0.003)
	[-0.544,-0.534]	[-0.498,-0.483]	[-0.514,-0.502]	[-0.531,-0.520]	[-0.548,-0.537]
INT_ref	-0.000***	-0.049***	-0.031***	-0.014***	0.004***
	(0.000)	(0.003)	(0.002)	(0.001)	(0.000)
	[-0.001,-0.000]	[-0.055,-0.043]	[-0.035,-0.028]	[-0.016,-0.012]	[0.003,0.004]
INT_med	0.005***	0.005***	0.005***	0.005***	0.005***
	(0.000)	(0.000)	(0.000)	(0.000)	(0.000)
	[0.005,0.006]	[0.005,0.006]	[0.005,0.006]	[0.005,0.006]	[0.005,0.006]
PIE	-0.018***	-0.018***	-0.018***	-0.018***	-0.018***
	(0.000)	(0.000)	(0.000)	(0.000)	(0.000)
	[-0.019,-0.017]	[-0.019,-0.017]	[-0.019,-0.017]	[-0.019,-0.017]	[-0.019,-0.017]
Prop. CDE	0.977***	0.889***	0.921***	0.952***	0.984***
	(0.001)	(0.005)	(0.003)	(0.001)	(0.001)
	[0.975,0.978]	[0.879,0.899]	[0.914,0.927]	[0.950,0.955]	[0.982,0.986]
Prop. INT_ref	0.001***	0.088***	0.057***	0.025***	-0.006***
	(0.000)	(0.005)	(0.003)	(0.002)	(0.000)
	[0.000,0.001]	[0.078,0.099]	[0.050,0.064]	[0.022,0.028]	[-0.007,-0.006]
Prop. INT_med	-0.010***	-0.010***	-0.010***	-0.010***	-0.010***
	(0.001)	(0.001)	(0.001)	(0.001)	(0.001)
	[-0.011,-0.009]	[-0.011,-0.009]	[-0.011,-0.009]	[-0.011,-0.009]	[-0.011,-0.009]
Prop. PIE	0.032***	0.032***	0.032***	0.032***	0.032***
	(0.001)	(0.001)	(0.001)	(0.001)	(0.001)
	[0.031,0.034]	[0.031,0.034]	[0.031,0.034]	[0.031,0.034]	[0.031,0.034]
OP med	0.022***	0.022***	0.022***	0.022***	0.022***
	(0.001)	(0.001)	(0.001)	(0.001)	(0.001)
	[0.021,0.024]	[0.021,0.024]	[0.021,0.024]	[0.021,0.024]	[0.021,0.024]
OP ati	-0.009***	0.078***	0.047***	0.015***	-0.016***
	(0.001)	(0.005)	(0.003)	(0.001)	(0.001)
	[-0.010,-0.008]	[0.069,0.088]	[0.041,0.052]	[0.013,0.017]	[-0.018,-0.014]
OP eliminated	0.023***	0.111***	0.079***	0.048***	0.016***
	(0.001)	(0.005)	(0.003)	(0.001)	(0.001)
	[0.022,0.025]	[0.101,0.121]	[0.073,0.086]	[0.045,0.050]	[0.014,0.018]
**Observations**	**503,656**	**503,656**	**503,656**	**503,656**	**503,656**

Note: Standard errors in parenthesis; 95% Confidence intervals in brackets; * p<0.10, ** p<0.05, *** p<0.01. TE=total effect; CDE=controlled direct effect; INT_ref=reference interaction; INT_med=mediated interaction; PIE=pure indirect effect; Prop. CDE=proportion controlled direct effect; Prop. INT_ref=proportion reference interaction; Prop. INT_med=proportion mediated interaction; prop. PIE=proportion pure indirect effect; OP med=overall proportion mediated; OP ati=overall proportion attributable to interaction; OP eliminated=overall proportion eliminated.

Supplementary Table 2: 4-Way decomposition results, 73 countries, reading test scores, PISA 2022

Effects	Closing Gap	Fixing M = 0	Fixing M = 0.2	Fixing M = 0.4	Fixing M = 0.6
TE	-0.525***	-0.525***	-0.525***	-0.525***	-0.525***
	(0.003)	(0.003)	(0.003)	(0.003)	(0.003)
	[-0.530,-0.519]	[-0.530,-0.519]	[-0.530,-0.519]	[-0.530,-0.519]	[-0.530,-0.519]
CDE	-0.512***	-0.468***	-0.484***	-0.500***	-0.516***
	(0.003)	(0.004)	(0.003)	(0.003)	(0.003)
	[-0.518,-0.507]	[-0.476,-0.461]	[-0.490,-0.478]	[-0.505,-0.495]	[-0.521,-0.511]
INT_ref	-0.000***	-0.044***	-0.028***	-0.013***	0.003***
	(0.000)	(0.003)	(0.002)	(0.001)	(0.000)
	[-0.000,-0.000]	[-0.050,-0.039]	[-0.032,-0.025]	[-0.014,-0.011]	[0.003,0.004]
INT_med	0.005***	0.005***	0.005***	0.005***	0.005***
	(0.000)	(0.000)	(0.000)	(0.000)	(0.000)
	[0.004,0.006]	[0.004,0.006]	[0.004,0.006]	[0.004,0.006]	[0.004,0.006]
PIE	-0.017***	-0.017***	-0.017***	-0.017***	-0.017***
	(0.000)	(0.000)	(0.000)	(0.000)	(0.000)
	[-0.018,-0.016]	[-0.018,-0.016]	[-0.018,-0.016]	[-0.018,-0.016]	[-0.018,-0.016]
Prop. CDE	0.977***	0.893***	0.923***	0.953***	0.984***
	(0.001)	(0.005)	(0.003)	(0.001)	(0.001)
	[0.975,0.978]	[0.882,0.903]	[0.917,0.930]	[0.951,0.956]	[0.982,0.986]
Prop. INT_ref	0.001***	0.085***	0.054***	0.024***	-0.006***
	(0.000)	(0.006)	(0.004)	(0.002)	(0.000)
	[0.000,0.001]	[0.073,0.096]	[0.047,0.061]	[0.021,0.027]	[-0.007,-0.005]
Prop. INT_med	-0.010***	-0.010***	-0.010***	-0.010***	-0.010***
	(0.001)	(0.001)	(0.001)	(0.001)	(0.001)
	[-0.011,-0.008]	[-0.011,-0.008]	[-0.011,-0.008]	[-0.011,-0.008]	[-0.011,-0.008]
Prop. PIE	0.032***	0.032***	0.032***	0.032***	0.032***
	(0.001)	(0.001)	(0.001)	(0.001)	(0.001)
	[0.030,0.034]	[0.030,0.034]	[0.030,0.034]	[0.030,0.034]	[0.030,0.034]
OP med	0.023***	0.023***	0.023***	0.023***	0.023***
	(0.001)	(0.001)	(0.001)	(0.001)	(0.001)
	[0.021,0.024]	[0.021,0.024]	[0.021,0.024]	[0.021,0.024]	[0.021,0.024]
OP ati	-0.009***	0.075***	0.045***	0.015***	-0.016***
	(0.001)	(0.005)	(0.003)	(0.001)	(0.001)
	[-0.010,-0.008]	[0.065,0.085]	[0.039,0.051]	[0.013,0.017]	[-0.018,-0.014]
OP eliminated	0.023***	0.107***	0.077***	0.047***	0.016***
	(0.001)	(0.005)	(0.003)	(0.001)	(0.001)
	[0.022,0.025]	[0.097,0.118]	[0.070,0.083]	[0.044,0.049]	[0.014,0.018]
**Observations**	**503,656**	**503,656**	**503,656**	**503,656**	**503,656**

Note: Standard errors in parenthesis; 95% Confidence intervals in brackets; * p<0.10, ** p<0.05, *** p<0.01. TE=total effect; CDE=controlled direct effect; INT_ref=reference interaction; INT_med=mediated interaction; PIE=pure indirect effect; Prop. CDE=proportion controlled direct effect; Prop. INT_ref=proportion reference interaction; Prop. INT_med=proportion mediated interaction; prop. PIE=proportion pure indirect effect; OP med=overall proportion mediated; OP ati=overall proportion attributable to interaction; OP eliminated=overall proportion eliminated.

Supplementary Figure 3: Proportion mediated (PIE) in 4-Way decomposition by country and PISA Test Score

Supplementary Figure 3 shows the proportion mediated (PIE) in a 4-Way decomposition by country and PISA test score for Math, Science, and Reading.

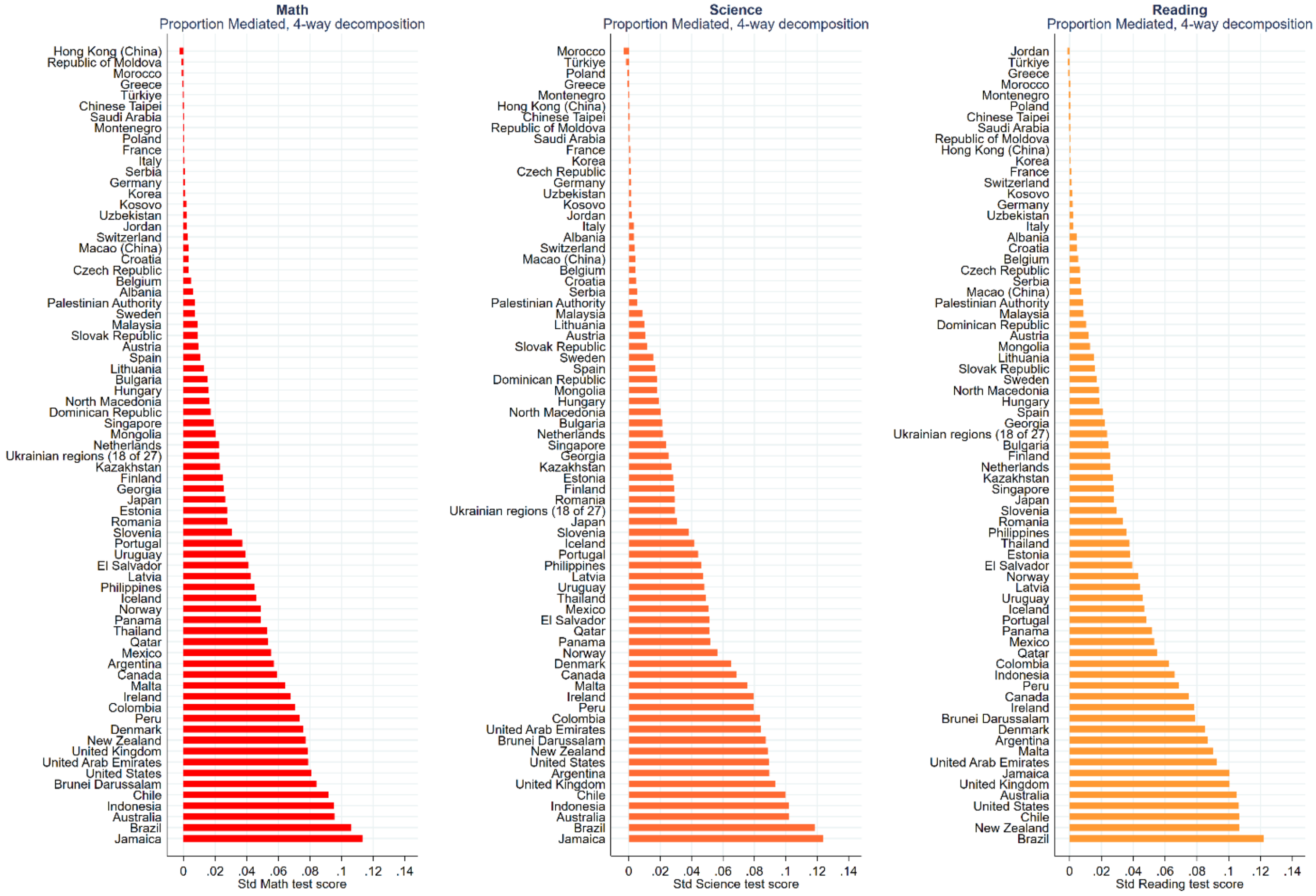

Country ranking from lowest to highest test scores

Supplementary Figure 4: Proportion mediated interaction and mediated interaction effect (INT_med) in 4-Way decomposition by country and PISA Test Score

Supplementary Figure 4 shows the proportion mediated interaction and the mediated interaction effect (INT_med) from a 4-Way decomposition by country and PISA test score for Math, Science, and Reading.

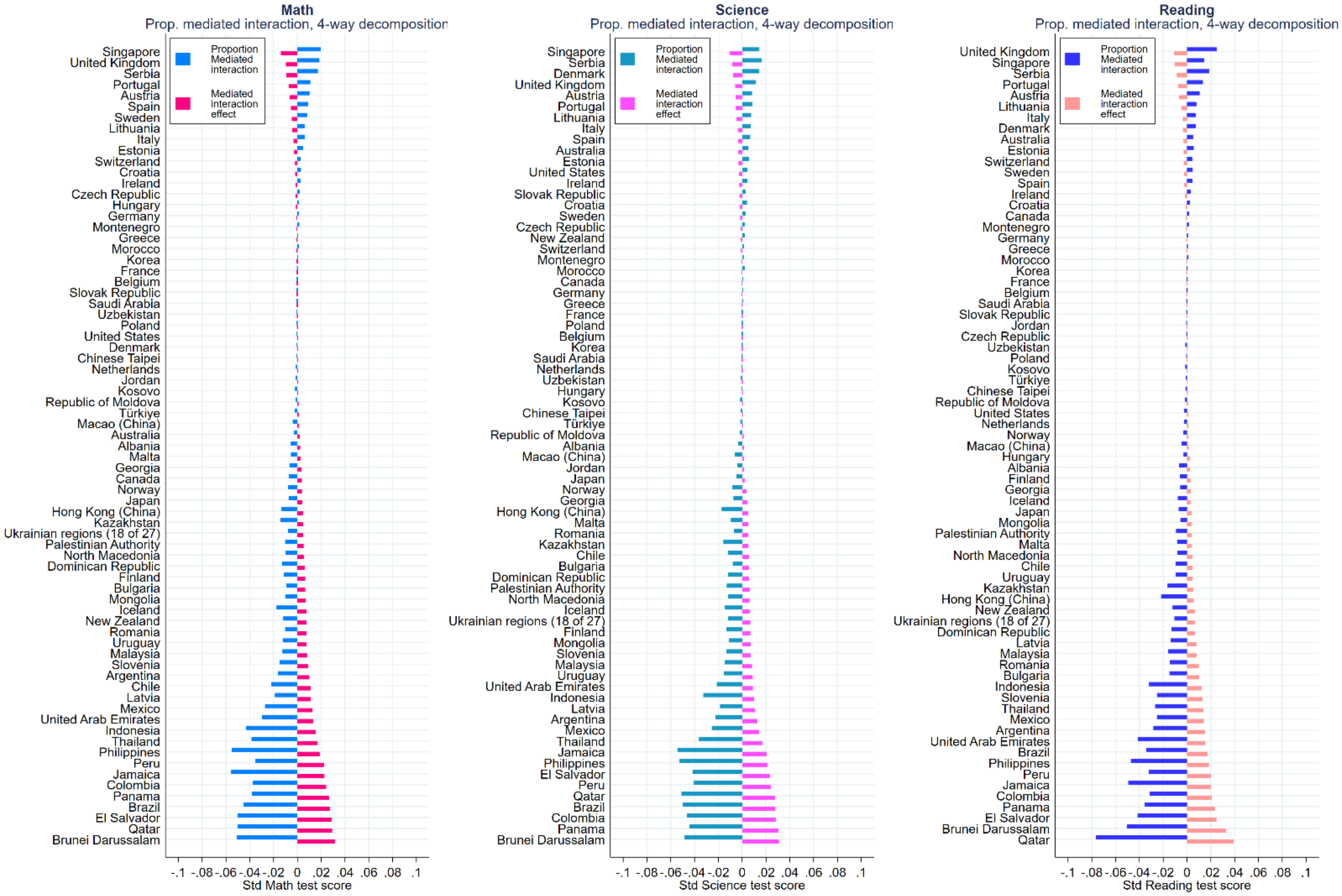

Country ranking from lowest to highest test scores

Additional Results without including the control variables in the Main Four-Way Decomposition model

Supplementary Table 3: 4-Way decomposition results without control variables, 73 countries, Math test scores, PISA 2022

Effects	Closing Gap	Fixing M = 0	Fixing M = 0.2	Fixing M = 0.4	Fixing M = 0.6
TE	-0.580***	-0.580***	-0.580***	-0.580***	-0.580***
	(0.003)	(0.003)	(0.003)	(0.003)	(0.003)
	[-0.586,-0.574]	[-0.586,-0.574]	[-0.586,-0.574]	[-0.586,-0.574]	[-0.586,-0.574]
CDE	-0.572***	-0.528***	-0.544***	-0.560***	-0.576***
	(0.003)	(0.004)	(0.004)	(0.003)	(0.003)
	[-0.578,-0.566]	[-0.537,-0.519]	[-0.551,-0.537]	[-0.566,-0.554]	[-0.582,-0.570]
INT_ref	0.000	-0.044***	-0.028***	-0.012***	0.004***
	(0.000)	(0.003)	(0.002)	(0.001)	(0.000)
	[-0.000,0.000]	[-0.051,-0.037]	[-0.032,-0.024]	[-0.014,-0.010]	[0.004,0.005]
INT_med	0.004***	0.004***	0.004***	0.004***	0.004***
	(0.000)	(0.000)	(0.000)	(0.000)	(0.000)
	[0.004,0.005]	[0.004,0.005]	[0.004,0.005]	[0.004,0.005]	[0.004,0.005]
PIE	-0.012***	-0.012***	-0.012***	-0.012***	-0.012***
	(0.000)	(0.000)	(0.000)	(0.000)	(0.000)
	[-0.013,-0.012]	[-0.013,-0.012]	[-0.013,-0.012]	[-0.013,-0.012]	[-0.013,-0.012]
Prop. CDE	0.986***	0.910***	0.938***	0.966***	0.994***
	(0.001)	(0.006)	(0.004)	(0.001)	(0.001)
	[0.985,0.987]	[0.899,0.921]	[0.931,0.945]	[0.963,0.969]	[0.991,0.996]
Prop. INT_ref	-0.000	0.076***	0.048***	0.020***	-0.007***
	(0.000)	(0.006)	(0.004)	(0.002)	(0.001)
	[-0.000,0.000]	[0.064,0.087]	[0.041,0.055]	[0.017,0.023]	[-0.009,-0.006]
Prop. INT_med	-0.008***	-0.008***	-0.008***	-0.008***	-0.008***
	(0.001)	(0.001)	(0.001)	(0.001)	(0.001)
	[-0.009,-0.006]	[-0.009,-0.006]	[-0.009,-0.006]	[-0.009,-0.006]	[-0.009,-0.006]
Prop. PIE	0.021***	0.021***	0.021***	0.021***	0.021***
	(0.001)	(0.001)	(0.001)	(0.001)	(0.001)
	[0.020,0.023]	[0.020,0.023]	[0.020,0.023]	[0.020,0.023]	[0.020,0.023]
OP med	0.014***	0.014***	0.014***	0.014***	0.014***
	(0.001)	(0.001)	(0.001)	(0.001)	(0.001)
	[0.013,0.015]	[0.013,0.015]	[0.013,0.015]	[0.013,0.015]	[0.013,0.015]
OP ati	-0.008***	0.068***	0.041***	0.013***	-0.015***
	(0.001)	(0.005)	(0.003)	(0.001)	(0.001)
	[-0.009,-0.006]	[0.058,0.079]	[0.034,0.047]	[0.011,0.015]	[-0.017,-0.013]
OP eliminated	0.014***	0.090***	0.062***	0.034***	0.006***
	(0.001)	(0.006)	(0.004)	(0.001)	(0.001)
	[0.013,0.015]	[0.079,0.101]	[0.055,0.069]	[0.031,0.037]	[0.004,0.009]
**Observations**	**503,656**	**503,656**	**503,656**	**503,656**	**503,656**

Note: Standard errors in parenthesis; 95% Confidence intervals in brackets; * p<0.10, ** p<0.05, *** p<0.01. TE=total effect; CDE=controlled direct effect; INT_ref=reference interaction; INT_med=mediated interaction; PIE=pure indirect effect; Prop. CDE=proportion controlled direct effect; Prop. INT_ref=proportion reference interaction; Prop. INT_med=proportion mediated interaction; prop. PIE=proportion pure indirect effect; OP med=overall proportion mediated; OP ati=overall proportion attributable to interaction; OP eliminated=overall proportion eliminated.

Supplementary Table 4: 4-Way decomposition results without control variables, 73 countries, Science test scores, PISA 2022

Effects	Closing Gap	Fixing M = 0	Fixing M = 0.2	Fixing M = 0.4	Fixing M = 0.6
TE	-0.552***	-0.552***	-0.552***	-0.552***	-0.552***
	(0.003)	(0.003)	(0.003)	(0.003)	(0.003)
	[-0.559,-0.546]	[-0.559,-0.546]	[-0.559,-0.546]	[-0.559,-0.546]	[-0.559,-0.546]
CDE	-0.544***	-0.497***	-0.514***	-0.531***	-0.548***
	(0.003)	(0.005)	(0.004)	(0.003)	(0.003)
	[-0.550,-0.537]	[-0.506,-0.488]	[-0.521,-0.507]	[-0.538,-0.525]	[-0.555,-0.542]
INT_ref	0.000	-0.046***	-0.029***	-0.012***	0.005***
	(0.000)	(0.003)	(0.002)	(0.001)	(0.000)
	[-0.000,0.000]	[-0.053,-0.040]	[-0.034,-0.025]	[-0.014,-0.011]	[0.004,0.005]
INT_med	0.005***	0.005***	0.005***	0.005***	0.005***
	(0.000)	(0.000)	(0.000)	(0.000)	(0.000)
	[0.004,0.005]	[0.004,0.005]	[0.004,0.005]	[0.004,0.005]	[0.004,0.005]
PIE	-0.013***	-0.013***	-0.013***	-0.013***	-0.013***
	(0.000)	(0.000)	(0.000)	(0.000)	(0.000)
	[-0.014,-0.013]	[-0.014,-0.013]	[-0.014,-0.013]	[-0.014,-0.013]	[-0.014,-0.013]
Prop. CDE	0.984***	0.900***	0.931***	0.961***	0.992***
	(0.001)	(0.006)	(0.004)	(0.002)	(0.001)
	[0.983,0.986]	[0.888,0.912]	[0.923,0.938]	[0.958,0.965]	[0.990,0.995]
Prop. INT_ref	-0.000	0.084***	0.053***	0.023***	-0.008***
	(0.000)	(0.006)	(0.004)	(0.002)	(0.001)
	[-0.000,0.000]	[0.072,0.097]	[0.046,0.061]	[0.019,0.026]	[-0.010,-0.007]
Prop. INT_med	-0.008***	-0.008***	-0.008***	-0.008***	-0.008***
	(0.001)	(0.001)	(0.001)	(0.001)	(0.001)
	[-0.010,-0.007]	[-0.010,-0.007]	[-0.010,-0.007]	[-0.010,-0.007]	[-0.010,-0.007]
Prop. PIE	0.024***	0.024***	0.024***	0.024***	0.024***
	(0.001)	(0.001)	(0.001)	(0.001)	(0.001)
	[0.023,0.026]	[0.023,0.026]	[0.023,0.026]	[0.023,0.026]	[0.023,0.026]
OP med	0.016***	0.016***	0.016***	0.016***	0.016***
	(0.001)	(0.001)	(0.001)	(0.001)	(0.001)
	[0.015,0.017]	[0.015,0.017]	[0.015,0.017]	[0.015,0.017]	[0.015,0.017]
OP ati	-0.008***	0.076***	0.045***	0.014***	-0.017***
	(0.001)	(0.006)	(0.003)	(0.001)	(0.001)
	[-0.010,-0.007]	[0.065,0.087]	[0.038,0.052]	[0.012,0.016]	[-0.019,-0.014]
OP eliminated	0.016***	0.100***	0.069***	0.039***	0.008***
	(0.001)	(0.006)	(0.004)	(0.002)	(0.001)
	[0.014,0.017]	[0.088,0.112]	[0.062,0.077]	[0.035,0.042]	[0.005,0.010]
**Observations**	**503,656**	**503,656**	**503,656**	**503,656**	**503,656**

Note: Standard errors in parenthesis; 95% Confidence intervals in brackets; * p<0.10, ** p<0.05, *** p<0.01. TE=total effect; CDE=controlled direct effect; INT_ref=reference interaction; INT_med=mediated interaction; PIE=pure indirect effect; Prop. CDE=proportion controlled direct effect; Prop. INT_ref=proportion reference interaction; Prop. INT_med=proportion mediated interaction; prop. PIE=proportion pure indirect effect; OP med=overall proportion mediated; OP ati=overall proportion attributable to interaction; OP eliminated=overall proportion eliminated.

Supplementary Table 5: 4-Way decomposition results without control variables, 73 countries, Reading test scores, PISA 2022

Effects	Closing Gap	Fixing M = 0	Fixing M = 0.2	Fixing M = 0.4	Fixing M = 0.6
TE	-0.528***	-0.528***	-0.528***	-0.528***	-0.528***
	(0.003)	(0.003)	(0.003)	(0.003)	(0.003)
	[-0.534,-0.522]	[-0.534,-0.522]	[-0.534,-0.522]	[-0.534,-0.522]	[-0.534,-0.522]
CDE	-0.520***	-0.474***	-0.491***	-0.508***	-0.524***
	(0.003)	(0.005)	(0.004)	(0.003)	(0.003)
	[-0.526,-0.514]	[-0.483,-0.466]	[-0.498,-0.484]	[-0.514,-0.501]	[-0.531,-0.518]
INT_ref	0.000	-0.046***	-0.029***	-0.012***	0.004***
	(0.000)	(0.003)	(0.002)	(0.001)	(0.000)
	[-0.000,0.000]	[-0.052,-0.039]	[-0.033,-0.025]	[-0.014,-0.010]	[0.004,0.005]
INT_med	0.005***	0.005***	0.005***	0.005***	0.005***
	(0.000)	(0.000)	(0.000)	(0.000)	(0.000)
	[0.004,0.005]	[0.004,0.005]	[0.004,0.005]	[0.004,0.005]	[0.004,0.005]
PIE	-0.013***	-0.013***	-0.013***	-0.013***	-0.013***
	(0.000)	(0.000)	(0.000)	(0.000)	(0.000)
	[-0.013,-0.012]	[-0.013,-0.012]	[-0.013,-0.012]	[-0.013,-0.012]	[-0.013,-0.012]
Prop. CDE	0.985***	0.899***	0.930***	0.962***	0.993***
	(0.001)	(0.006)	(0.004)	(0.002)	(0.001)
	[0.983,0.986]	[0.886,0.911]	[0.923,0.938]	[0.959,0.965]	[0.991,0.996]
Prop. INT_ref	-0.000	0.086***	0.055***	0.023***	-0.008***
	(0.000)	(0.007)	(0.004)	(0.002)	(0.001)
	[-0.000,0.000]	[0.073,0.099]	[0.046,0.063]	[0.020,0.027]	[-0.010,-0.007]
Prop. INT_med	-0.009***	-0.009***	-0.009***	-0.009***	-0.009***
	(0.001)	(0.001)	(0.001)	(0.001)	(0.001)
	[-0.010,-0.007]	[-0.010,-0.007]	[-0.010,-0.007]	[-0.010,-0.007]	[-0.010,-0.007]
Prop. PIE	0.024***	0.024***	0.024***	0.024***	0.024***
	(0.001)	(0.001)	(0.001)	(0.001)	(0.001)
	[0.022,0.025]	[0.022,0.025]	[0.022,0.025]	[0.022,0.025]	[0.022,0.025]
OP med	0.015***	0.015***	0.015***	0.015***	0.015***
	(0.001)	(0.001)	(0.001)	(0.001)	(0.001)
	[0.014,0.016]	[0.014,0.016]	[0.014,0.016]	[0.014,0.016]	[0.014,0.016]
OP ati	-0.009***	0.078***	0.046***	0.014***	-0.017***
	(0.001)	(0.006)	(0.004)	(0.001)	(0.001)
	[-0.010,-0.007]	[0.066,0.089]	[0.039,0.053]	[0.012,0.017]	[-0.020,-0.014]
OP eliminated	0.015***	0.101***	0.070***	0.038***	0.007***
	(0.001)	(0.006)	(0.004)	(0.002)	(0.001)
	[0.014,0.017]	[0.089,0.114]	[0.062,0.077]	[0.035,0.041]	[0.004,0.009]
**Observations**	**503,656**	**503,656**	**503,656**	**503,656**	**503,656**

Note: Standard errors in parenthesis; 95% Confidence intervals in brackets; * p<0.10, ** p<0.05, *** p<0.01. TE=total effect; CDE=controlled direct effect; INT_ref=reference interaction; INT_med=mediated interaction; PIE=pure indirect effect; Prop. CDE=proportion controlled direct effect; Prop. INT_ref=proportion reference interaction; Prop. INT_med=proportion mediated interaction; prop. PIE=proportion pure indirect effect; OP med=overall proportion mediated; OP ati=overall proportion attributable to interaction; OP eliminated=overall proportion eliminated.

C. Estimation Details for Continuous Outcome & Binary Mediator

Defining Y as a continuous outcome, A as binary treatment and M as binary mediator, under assumptions (i)-(iv) and correct specification of the regression models for Y and M:

[1]

$$E[Y \mid a, m, c] = \theta_0 + \theta_1 a + \theta_2 m + \theta_3 am + \theta_4' c$$
$$\text{logit}\,\{P(M = 1 \mid a, c)\} = \beta_0 + \beta_1 a + \beta_2' c.$$

Valeri and VanderWeele (2013) show that the average controlled direct effect (CDE) and the average pure indirect effect (PIE) are given by:

[2]

$$E[CDE(m^*) \mid c] = (\theta_1 + \theta_3 m^*)(a - a^*)$$
$$E[PIE \mid c] = (\theta_2 + \theta_3 a^*)\left\{\frac{\exp[\beta_0 + \beta_1 a + \beta_2' c]}{1 + \exp\,[\beta_0 + \beta_1 a + \beta_2' c]} - \frac{\exp[\beta_0 + \beta_1 a^* + \beta_2' c]}{1 + \exp\,[\beta_0 + \beta_1 a^* + \beta_2' c]}\right\}$$

The reference interaction (INT_ref) is given by the difference between the pure direct effect and the controlled direct effect, which were both given by Valeri and VanderWeele:

[3]

$$E\left[INT_{ref}(m^*) \mid c\right] = \{\theta_1(a-a^*)\} + \{\theta_3(a-a^*)\}\frac{\exp[\beta_0+\beta_1 a^*+\beta_2' c]}{1+\exp[\beta_0+\beta_1 a^*+\beta_2' c]} - (\theta_1+\theta_3 m^*)(a-a^*)$$
$$= \theta_3(a-a^*)\left(\frac{\exp[\beta_0+\beta_1 a^*+\beta_2' c]}{1+\exp[\beta_0+\beta_1 a^*+\beta_2' c]} - m^*\right)$$

The mediated interaction (INT_med) is given by the difference between the total indirect effect and the pure indirect effect:

[4]

$$E[INT_{\text{med}} \mid c] = (\theta_2+\theta_3 a)\left\{\frac{\exp[\beta_0+\beta_1 a+\beta_2' c]}{1+\exp[\beta_0+\beta_1 a+\beta_2' c]} - \frac{\exp[\beta_0+\beta_1 a^*+\beta_2' c]}{1+\exp[\beta_0+\beta_1 a^*+\beta_2' c]}\right\}$$
$$-(\theta_2+\theta_3 a^*)\left\{\frac{\exp[\beta_0+\beta_1 a+\beta_2' c]}{1+\exp[\beta_0+\beta_1 a+\beta_2' c]} - \frac{\exp[\beta_0+\beta_1 a^*+\beta_2' c]}{1+\exp[\beta_0+\beta_1 a^*+\beta_2' c]}\right\}$$
$$= \theta_3(a-a^*)\left\{\frac{\exp[\beta_0+\beta_1 a+\beta_2' c]}{1+\exp[\beta_0+\beta_1 a+\beta_2' c]} - \frac{\exp[\beta_0+\beta_1 a^*+\beta_2' c]}{1+\exp[\beta_0+\beta_1 a^*+\beta_2' c]}\right\}$$

Supplementary References

Valeri, Linda, and Tyler J. Vanderweele. 2013. "Mediation Analysis Allowing for Exposure-Mediator Interactions and Causal Interpretation: Theoretical Assumptions and Implementation with SAS and SPSS Macros." *Psychological Methods* 18:137–50. doi: 10.1037/a0031034.
